# Beyond Antibiotics: Photo/Sonodynamic Approaches for Bacterial Theranostics

**DOI:** 10.1007/s40820-020-00485-3

**Published:** 2020-07-10

**Authors:** Xin Pang, Dengfeng Li, Jing Zhu, Jingliang Cheng, Gang Liu

**Affiliations:** 1 Henan Key Laboratory of Functional Magnetic Resonance Imaging and Molecular Imaging, Department of Magnetic Resonance Imaging, The First Affiliated Hospital of Zhengzhou University, 450052 Zhengzhou, People’s Republic of China; 2 State Key Laboratory of Molecular Vaccinology and Molecular Diagnostics and Center for Molecular Imaging and Translational Medicine, School of Public Health, Xiamen University, 361102 Xiamen, People’s Republic of China; 3 Amoy Hopeful Biotechnology Co., Ltd, 361027 Xiamen, People’s Republic of China

**Keywords:** Photodynamic therapy, Sonodynamic therapy, Theranostics, Multidrug resistant, Reactive oxygen species

## Abstract

Rapid evolution and propagation of multidrug resistance among bacterial pathogens are outpacing the development of new antibiotics, but antimicrobial photodynamic therapy (aPDT) provides an excellent alternative. This treatment depends on the interaction between light and photoactivated sensitizer to generate reactive oxygen species (ROS), which are highly cytotoxic to induce apoptosis in virtually all microorganisms without resistance concern. When replacing light with low-frequency ultrasonic wave to activate sensitizer, a novel ultrasound-driven treatment emerges as antimicrobial sonodynamic therapy (aSDT). Recent advances in aPDT and aSDT reveal golden opportunities for the management of multidrug resistant bacterial infections, especially in the theranostic application where imaging diagnosis can be accomplished facilely with the inherent optical characteristics of sensitizers, and the generated ROS by aPDT/SDT cause broad-spectrum oxidative damage for sterilization. In this review, we systemically outline the mechanisms, targets, and current progress of aPDT/SDT for bacterial theranostic application. Furthermore, potential limitations and future perspectives are also highlighted.

## Highlights

- Recent advances in bacterial theranostics using antimicrobial photo/sonodynamic therapy (aPDT/SDT) are summarized in this review.
- The inherent optical characteristics of photo/sonosensitizers facilely enable imaging diagnosis of bacterial infections.
- Reactive oxygen species as the killing effector of aPDT/SDT cause broad-spectrum damage for sterilization with no concern about antibiotic resistance.

## Graphical abstract

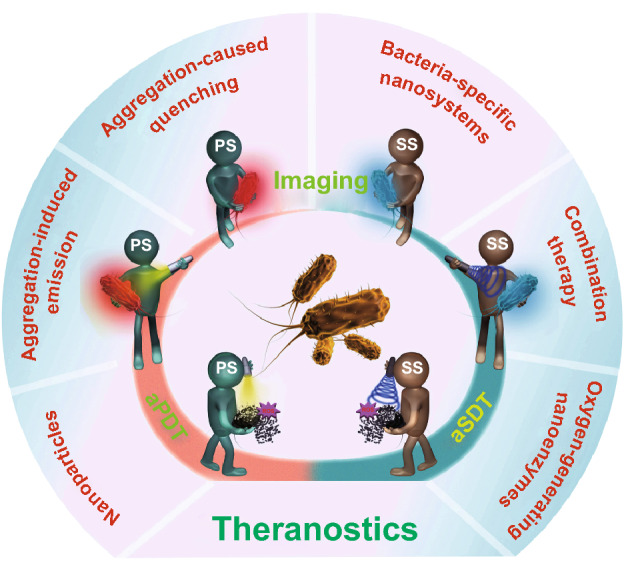

## Introduction

The worrying increase of infections caused by pathogenic bacterial strains, especially multidrug resistant (MDR) species, is an ever-growing global crisis in human medicine. Such MDR bacteria are able to propagate very quickly not only in the hospital facilities but also in the community, which exact a staggering epidemiological and economic burden on the health care system [1]. More alarmingly, the poor capability for accurate and timely infection diagnosis by current clinical technologies further aggravates the resistance situation [2]. It is estimated that unless effective action is taken, the burden of deaths from antimicrobial resistance could balloon to 10 million lives each year by 2050, far more than the number of people dying from cancer [3]. We are now approaching a post-antibiotic era in which normal infections or minor injuries are no longer treatable. However, contrary to the inexorable emergence of antibiotic resistance, the development rate of novel-acting antibiotic has greatly plummeted over the past 30 years and many pharmaceutical companies even abandoned antibiotic discovery programs. Plagued by the drying antibiotic pipeline, alternative therapeutics to combat MDR bacteria, particularly to block the existing resistance evolution and prevent new resistance emergence, are urgently required in the battle of antimicrobial stewardship.

Reliable infection detection is the fundamental first step for therapeutic interventions and can lead to a more precise and targeted bacterial management. However, in addition to multidrug resistance, another major obstacle for current antimicrobial stewardship is the inability to diagnose bacterial infections with accuracy and sensitivity. Current bacterial determination mainly relies on conventional tissue biopsies and microbiological analysis, which are labor-intensive, time-consuming and difficult for on-site diagnosis [4]. In clinic, many infectious diseases are still diagnosed by their clinical presentation, but the clinical symptoms and signs of clinical manifestations are often unobvious in the early stage [5]. Although newly developed ^67^Ga-citrate, ^18^F-FDG, and radiolabeled leukocytes have been marketed as tracers for infection imaging and provide good sensitivity for early-stage diagnosis, these leukocyte and metabolic imaging methods are inherently non-specific, still failing to distinguish bacterial infections from other inflammatory disorders [6]. All of these prompt the continuous increase of misdiagnosis and subsequent mismanagement. On the contrary, rapid, sophisticated, and highly sensitive diagnostic technologies can guarantee the implementation of correct therapeutic regimen, avoid the inordinate prescription of drugs, and, in turn, reduce the emergence of antimicrobial resistance [7, 8]. Given this, management of bacterial infections, especially those caused by MDR strains, demands a multipronged strategy that includes not only the development of novel-acting therapeutics, but also exploiting efficient diagnosis and discrimination techniques, so as to ensure the accomplishment of targeted and rational infection control principles.

**Fig. 1:**
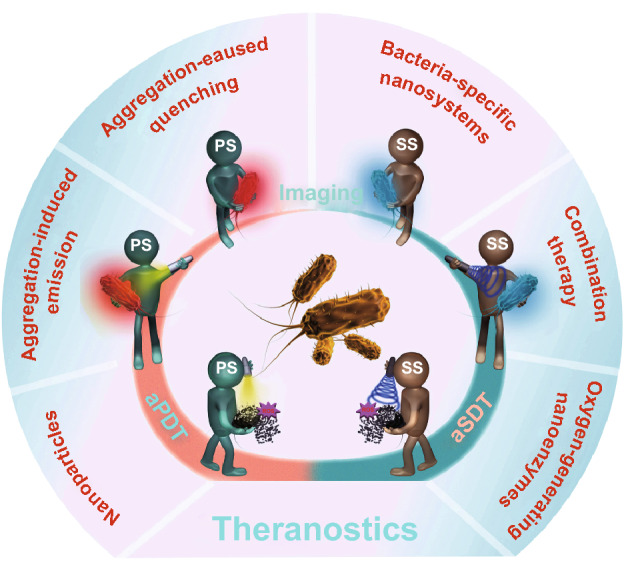
Schematic illumination of aPDT and aSDT for bacterial theranostic application. Bacterial imaging can be accomplished facilely with the inherent optical characteristics of photo/sonosensitizers, and the generated ROS of aPDT/SDT cause broad-spectrum oxidative damage for sterilization. *PS* photosensitizer, *SS* sonosensitizer

As physical therapeutics has come of age, we have witnessed a great transformation in the quest for antibacterial innovations. Much focus has turned toward physical (e.g., heat [9, 10], X-ray irradiation [11], and high-pressure [12]) inactivation of microbial cells because they are amenable to MDR pathogens and typically have less potential to induce resistance. Comparatively, light-driven photodynamic therapy presents an ever-attractive methodology against bacterial infections by virtue of its intrinsic safety and noninvasiveness [13]. Antimicrobial photodynamic therapy (aPDT) is based on the photodynamic therapy concept that mainly depends on three essential components, i.e., specific wavelength of light, a photoactivated sensitizer termed as photosensitizer, and molecular oxygen. Taken individually, none of them is harmful; only together they can activate a photochemical reaction to produce highly cytotoxic reactive oxygen species (ROS) [14]. If replacing light with low-intensity and frequency ultrasound to activate sensitizers, a novel physics-driven treatment emerges. Originating from aPDT, antimicrobial sonodynamic therapy (aSDT) is dependent on the interaction between ultrasonic wave and sonosensitizer to generate ROS and usually shares the same sensitizers with aPDT [15]. Owing to non-specific action mechanism and low mutagenic potential of ROS [16], both aPDT and aSDT are potent for virtually all microorganisms without resistance concern. Further benefiting from the noninvasiveness and site-confined irradiation of light or ultrasound, the potential systemic toxicity that frequently troubles conventional antibiotic therapy is promisingly avoided. If needed, the ROS-mediated aPDT/SDT also allows repeated treatments, and this process rarely induces bacterial resistance [17–19]. More importantly, it is a great bonus that most of photo/sonosensitizers possess inherent optical characteristics and therefore can be used as imaging probe to optically diagnose bacterial infections. Such facile and fast diagnosis technique is expected to alleviate current diagnosis dilemma. By integrating of therapeutic and diagnostic capability in a single system, the aPDT/SDT, serving as a theranostic platform (Fig. 1), provides golden opportunities for improving therapeutic outcomes and preserving our ability to implement and optimize current antimicrobial stewardship. In this review, we systemically outline the mechanisms, targets, and recent progress of aPDT/SDT for bacterial theranostic application. Furthermore, potential limitations and future perspectives are also highlighted.

## Mechanism of aPDT/SDT

**Fig. 2:**
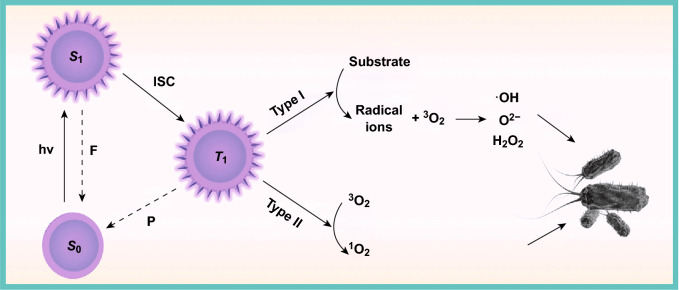
Photochemical processes involved in photodynamic inactivation of bacteria

In practice, photosensitizers can be administrated to the bacteria-infected subjects, either systemically, locally, or topically to the skin. At predefined time-interval that allows sufficient photosensitizers to concentrate, the lesion loci are irradiated with specific wavelength of laser (400–700 nm). After absorbing light photons, the photosensitizer will be excited from a ground state (*S*_0_) to a singlet state (*S*_1_). Because of strong instability, such excited-state electrons undergo an intersystem crossing and therefore convert into a more stable long-lived electronically triplet state (*T*_1_). In this process, excess energy is emitted as fluorescence which can be clinically exploited for imaging and photodetection. The *T*_1_ photosensitizer can experience two kinds of photochemical reactions (Fig. 2) [20]. First, it may transfer a hydrogen atom or electron to surrounding oxygen molecule, generating oxygenated radicals, including hydroxyl radicals (·OH), superoxide anion radical (O^2−^), as well as hydrogen peroxide (H_2_O_2_). This process is defined as Type I reaction. While in Type II procedure, the *T*_1_ state photosensitizer reacts directly with the triplet ground state oxygen (^3^O_2_) to form a highly bioactive singlet-state oxygen (^1^O_2_) [21]. Both of reactions occur simultaneously in one photodynamic case, and the ratio between Type I and Type II reactions is dependent on many factors, such as the type of photosensitizer molecules, the concentration of surrounding oxygen and substrate, and the binding ability of photosensitizer to substrate. Generally, ROS generation from Type II chemistry is fairly more simple than that from Type I at the mechanistic level, singlet oxygen therefore is believed to be the key of photodynamic activity for most photosensitizers [20].

As for aSDT, the exact working mechanism is still under debate, although its therapeutic efficacy has been well documented. It is widely accepted that ROS are the predominant killing effector of aSDT. In the process of ROS generation upon ultrasound irradiation, effective ultrasonic transfer through liquids/tissue fluids media is paramount. The interaction of ultrasound with aqueous environments results in a unique phenomenon known as acoustic cavitation which involves nucleation, growth, and the implosive collapse of gas-filled bubbles under appropriate ultrasound conditions [22]. Currently, two primary cavitation phenomena are proposed to activate sonosensitizer for producing ROS: sonoluminescence and pyrolysis. The concept of sonoluminescence refers to a process where light is briefly emitted via rapid energy release from bubble implosion during acoustic cavitation [23]. In lieu of sonoluminescence, sonochemical pyrolysis means the localized temperature and pressure elevated by inertial cavitation process, which further breaks apart the sonosensitizer to generate free radicals [24]. Accurate characterizations of ROS are beneficial in forecasting the therapeutic performances of aSDT in vitro and in vivo. Electron spin resonance (ESR) spin-trapping method is the most prominent analytical technique for cytotoxic ·OH and ^1^O_2_. Biochemical assays using fluorescent, luminescent, and colorimetric as detecting probes also provide useful tools for visualizing the quantitative changes of ROS. Typically, the sonication parameters adopted in aSDT are carried out within 0.5–2.0 MHz at an intensity of 0.5–3.0 W/cm^2^, and the irradiation time lasts 1–30 min [25]. Although increased ultrasound intensity and time are able to improve ROS production, the thermal or mechanical effects will strengthen simultaneously, causing undesirable damages to non-target cells.

## Targets of aPDT/SDT

Different classes of bacteria show a wide variation in the cellular structure and organization, which significantly influences the interaction of exogenous sensitizers with bacterial targets. In general, Gram-positive bacteria possess a cell wall comprised of teichoic acids, protein, and polysaccharides. They are organized in cross-linked and multiple peptidoglycan layers (20–80 nm) that confer a degree of porosity to allow sensitizer penetration into bacterial cell. In Gram-negative bacteria, the presence of an intricate outer membrane, surrounding a thinner peptidoglycan layer (2–7 nm), creates an impermeable barrier to sensitizers. The outer membrane consists of a phospholipid bilayer in the inner leaflet and various glycolipids in the outer leaflet, mainly lipopolysaccharides and porins. All of these impart structural integrity and protect the membrane from attacks by antibacterial agents [26]. Compared with Gram-negative species, their Gram-positive counterparts are much more susceptible to aPDT/SDT because of the thick but porous peptidoglycan layer for sensitizer entry. Both of bacterial species show an overall negatively charged cell surface. Such anionic envelope acts as an electro-attractive scaffold for cationic sensitizers that are more efficiently bound to and internalized by bacteria [27].

Due to short lifetimes and mobility of ROS, the sensitizers are preferable to penetrate or at least bind to bacterial cell wall for maximum oxidative damage following aPDT/SDT [28]. The ROS generated by sensitizer molecules may interact with different cellular components based on their affinities for these targets. In general, three putative bacterial targets have been proposed, including the cell membrane phospholipids, essential proteins, and nucleic acids. Comparatively, membrane proteins are considered as the preferred targets for photo/sonodynamic oxidation, not only due to their vital functions in bacteria [29], but also because they are abundant on the bacterial surface and able to quickly react with ROS after binding with exogenous sensitizers [30]. By damaging such targets, considerable morphological and functional changes of microbial cells are induced by aPDT/SDT. Morphological damages mainly contain the alteration of the mesosome structure. Direct destruction of bacterial cell wall and inner membrane will break membrane integrity, resulting in the leakage of cytoplasmic contents and subsequent inactivation of membrane transport system. Functional alterations are generally caused by disorder of membrane potential, loss of protein and enzyme activities, and inhibition of metabolic processes (e.g., DNA replication, glucose transport) [31]. In most cases, those two types of changes occur simultaneously. For instance, oxidative modification of component lipids alters membrane fluidity and organization as well as membrane protein function that, when extensive enough, culminate in cell death.

## PDT-Mediated Bacterial Theranostics

In the area of luminescence research, there is a common photophysical phenomenon called aggregation-caused quenching (ACQ): Luminophores exhibit bright emission in the mono-dispersed state but weakened or even quenched emission in the aggregated state. Such ACQ effect is frequently observed in traditional photosensitizers that have a rigid structure such as, porphyrin, phthalocyanine, and phenothiazinium. With concentration increasing or in the presence of unfavorable external environment, these photosensitizers tend to aggregate, while their emissions are attenuated sharply [32]. Interestingly, another photophysical phenomenon associated with luminophore aggregation is aggregation-induced emission (AIE). Opposite to the ACQ process, luminogens with AIE characteristics (AIEgens) are non-emissive in the dissolved state but become strongly fluorescent upon aggregation owing to the restriction of intramolecular motion [33]. What is more, with intelligent molecular designing, some AIE-based molecules were reported to provide an increased ability of ROS generation in the aggregated state, which can potentially serve as novel photosensitizers for photodynamic inactivation of pathogenic microbes. Based on such different photophysical phenomenon, the photosensitizers can be divided into two categories: ACQ-based photosensitizers and AIE-based photosensitizers. Apart from these small molecular photosensitizers, nanoparticles with photosensitive activity have also been envisioned as potential candidates. Here, we summarize current advances in PDT-mediated bacterial theranostics in the following discussion.

### Photosensitizers Based on ACQ

Recently, a large number of ACQ photosensitizers have been developed and investigated for aPDT; most of them are porphyrins and phthalocyanines, known as second-generation photosensitizers. In contrast, the development of photosensitizers which are not based on tetrapyrrole macrocycle framework is relatively less extensive, but structural modification of phenothiaziniums [34], hypericins [35], and rose bengals [36] allows a regulation of photodynamic activity to an interesting degree. As for aqueous solubility, the majority of ACQ photosensitizers are inherently hydrophobic. They are very prone to aggregate in the physiological condition, further lowering the water solubility and ROS production of photosensitizers. How to solve this issue is a vital task. Although the history, current status, and future prospects of ACQ photosensitizers for aPDT have been presented in the literature [37, 38], most of these articles only focus on the therapeutic application. In this section, a systemic overview of ACQ photosensitizers in bacterial imaging and eradication was provided.

#### Porphyrins

**Fig. 3:**
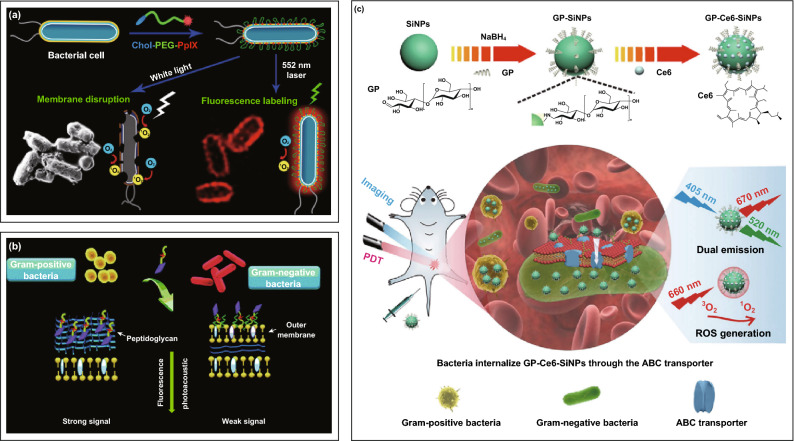
**a** Representation of aPDT-mediated labeling and killing of *Escherichia coli* (*E. coli*) by cholesterol-modified PpIX (Chol-PEG-PpIX) upon light irradiation [46]. Copyright 2017 American Chemical Society. **b** Schematic of polyarginine-purpurin conjugates for different photodynamic inactivation and imaging on Gram-positive and Gram-negative bacteria [48]. Copyright 2016 Royal Society of Chemistry. **c** Design of multifunctional nanoplatform with multiple emission signals for effective detection and photodynamic treatment of bacterial infections [49]. Copyright 2019 Creative commons

Porphyrins are the commonly used ACQ photosensitizers for aPDT due to their efficient light absorptivity, high quantum yield, facile synthesis, and chemical diversity (i.e., easy modification and functionalization) [39]. In the process of aPDT, the number of charges carried by porphyrin photosensitizer is closely related to its bactericidal efficacy. As we know, bacteria are typically more anionic than mammalian cells. Therefore, a cationic charge of photosensitizer can facilitate the effective electrostatic interaction between photosensitizer and bacterial cells, resulting in potent photoinactivation of bacterial strains [40]. To make porphyrins positively charged, one possible method is to introduce cationic substituents, such as pyridine and imidazolium [41]. Comparatively, a more versatile approach that integrates porphyrin photosensitizers with positively charged polymers (i.e., poly(allylamine hydrochloride) [42], polyethylenimine [43], and chitosan [44]) is widely adopted to ensure strong affinity with bacterial surface. However, most positive-charged materials were found to be highly toxic to mammalian cells, particularly red blood cells, which may cause severe hemolysis and immunogenic reactions [45]. Alternatively, diverse amphiphilic porphyrin conjugates were designed to achieve safe and effective bacterial surface engineering. It has been reported that cholesterol can significantly promote the hydrophobic anchoring of photosensitizer onto bacterial surface. Therefore, an effective labeling and photoinactivating both Gram-negative and Gram-positive bacteria were accomplished through the conjugation of cholesterol with photosensitizer protoporphyrin IX (PpIX) (Fig. 3a) [46]. Further depending on different cell envelope structures that Gram-negative bacteria have a unique lipopolysaccharide (LPS) moiety in the outer membrane but Gram-positive species have a peptidoglycan network outside of the plasma membrane, a LPS binding peptide-modified PpIX exhibited specific fluorescent imaging and photoinactivation of Gram-negative bacteria [47], while stronger photoacoustic imaging and photodynamic killing on Gram-positive strains were achieved by a polyarginine-purpurin derivate via selectively inserting into bacterial porous peptidoglycan network (Fig. 3b) [48]. The porphyrin conjugates-mediated cell engineering offers unprecedented opportunities to manipulate the bacterial surface, regulate their fate or function, as well as develop novel theranostics for antibacterial stewardship. Despite achieving impressive results, the development of simple and specific affinity conjugates that can selectively direct porphyrins to the bacterial cell surface still remains challenges, since the current approaches potentially encounter several obstacles including non-specific aggregation, tedious sample treatment, possible immune response, or less cellular penetration. Further optimization and new methods exploitation are required in the future.

Most of currently used photosensitizers only possess a single emission under a single excitation, and their single-emission signals are therefore easily influenced by the fluctuation of local photosensitizer concentration. Accordingly, these sensitizers-mediated imaging systems are always difficult to detect the bacterial infections efficiently or accurately, especially at low concentrations. Meanwhile, intricate infection microenvironment also poses an important threat to the single-emission imaging diagnosis. In this regard, the combination of multiple emission signals is a promising approach to synergistically realize precise and reliable bacterial detection, and possibly reduce the working concentration of imaging agents. As a typical example (Fig. 3c), a novel-acting nanoplatform was constructed using fluorescent silicon nanoparticles (SiNPs) with a glucose polymer (GP) modification and photosensitizer chlorin e6 (Ce6) loading [49]. By virtue of GP-mediated ATP-binding cassette transporter pathway, the nanoparticles were rapidly internalized into both Gram-negative and Gram-positive bacteria. Coupled with intrinsic green and red fluorescence from SiNPs and Ce6, respectively, the dual-emission imaging could efficiently track infections, allowing the limit of in vivo bacterial detection as few as 10^5^ colony-forming units (CFU). Upon laser exposure (660 nm, 12 mW cm^−2^, 40 min), the photodynamic antibacterial efficiency of nanoparticles is up to 98% against *Staphylococcus aureus* (*S. aureus*) and 96% against *Pseudomonas aeruginosa*. Due to highly integrating bacteria-specific recognition, ultrasensitive dual-emission imaging, and efficient photoinactivation of broad-spectrum bacteria, this design indicates great potential in bacterial theranostic application.

#### Phthalocyanines

**Fig. 4:**
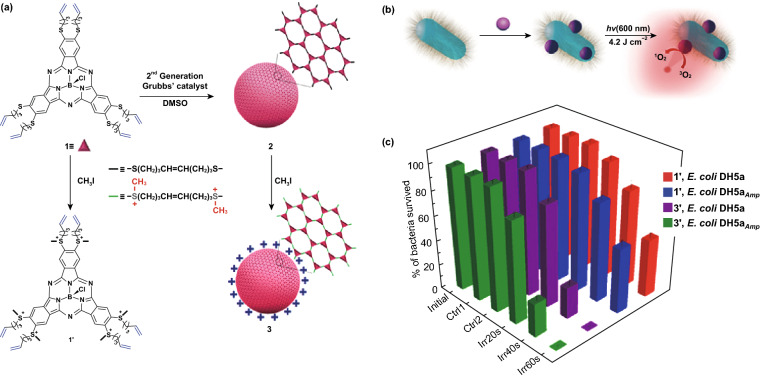
**a** Preparation of SubPc nanosphere. **b** Schematic illustration of SubPc nanosphere for bacterial labeling and photoinactivation. **c** Photodynamic killing of SubPc nanosphere on *E. coli* DH5a and the ampicillin-resistant strain *E. coli* DH5aAmp. Initial: original bacteria count. Ctrl 1: dark-toxicity experiments. Ctrl 2: phototoxicity experiments. Irr20s, Irr40s, and Irr60s: the bacterial suspension after photosensitizer incubation was irradiated with a laser for 20 s, 40 s, or 60 s, respectively [57]. Copyright 2015 Wiley-VCH

Phthalocyanine photosensitizers are aromatic macrocycle compounds. Different from porphyrins composed of secondary amine-connected isoindole subunits, phthalocyanines contain methane-interbridged tetrapyrrols, shifting the maximum absorption to longer wavelengths (typically > 660 nm) [50]. Compared with short wavelengths, long wavelengths display better tissue penetration and are more feasible for deep-seated infections. When imaging in vivo, these phthalocyanines with long-wavelength excitation can potently overcome the tissue absorption and scattering, enabling more reliable infection detection. Further coupled with high phototoxicity and stability toward self-oxidation, phthalocyanines have been explored as ideal photosensitizers for bacterial theranostics. Inevitably, the inherent hydrophobicity makes these molecules liable to aggregate in physiological conditions. To date, multiple approaches have been undertaken to overcome this limitation. One facile strategy is the introduction of bulky substituents on either peripheral or axial positions of the phthalocyanine macrocycle [51–53]. Thereinto, axially siloxane-functionalized phthalocyanines (Si^IV^-PCs) with low dark toxicity have been approved by US Food and Drug Administration (FDA) for PDT [49]. Further in-depth studies on axially substituted Si^IV^-PCs indicated that positive charge-mediated targeting using dicationic ammonium groups allowed the photosensitizer for fluorescence labeling and photodynamic inactivation of broad-spectrum bacteria, while neutral Si^IV^-PCs affected the Gram-positive cells only [54]. Stemming from the cone-shaped structure and 14-π-electron aromatic conjugated system, subphthalocyanines (SubPcs) as a class of phthalocyanine derivatives possess a longer triplet excited-state lifetime than Pcs, resulting in a higher quantum yield for ROS generation (0.67 for SubPcs and 0.52 for Pcs) [55, 56]. To improve their water dispersity and bacterial targeting, Kim’s group synthesized a covalently connected hollow SubPc nanosphere and modified positive charges on its surface (Fig. 4a) [57]. As expected, SubPc-based nanomaterials exhibited great ability to target, image, and photodynamic inactivate antibiotic-resistant *E. coli* bacteria with more than 99% potency, even at a light dose as low as 4.2 J cm^−2^ and a drug concentration of 10 nM only (Fig. 4b, c). Apart from the chemical substituents and using nanotechnology, several other strategies are also being explored to circumvent the aggregation issue of phthalocyanines. Particularly, the covalent attachment of phthalocyanines with hydrophilic biomolecules capable of lesion recognition is now dominating targeted photodynamic therapy and imaging. This methodology can not only improve the water solubility of phthalocyanines, but also make them disease selective. Although it has been widely applied in cancer management, the first example for bacterial theranostics is still awaited.

#### Phenothiaziniums

Phenothiazinium photosensitizers, mainly including methylene blue (MB), toluidine blue O, and azure dyes, are a class of first-generation photosensitizers, which have been investigated in aPDT for nearly 80 years. These photosensitizers are commonly amphipathic planar molecules that contain one intrinsic quaternary nitrogen atom. By virtue of intrinsic cationic property, phenothiaziniums can strongly bind with bacteria, showing photocytotoxicity against a broad spectrum of microorganisms, such as *S. aureus*, *E. coli*, *Pseudomonas aeruginosa*, *Acinetobacter baumannii*, and *Streptococci* [27]. It is pharmacodynamically interesting that this class of photosensitizers can be employed as inherent antibacterial compounds, also under dark conditions [58]. With respect to PDT, the photodynamic activity of phenothiaziniums mostly occurs via the Type I mechanism. To date, MB as the most well-known paradigm of phenothiaziniums has received regulatory approval to treat dental infectious diseases, such as periodontitis and caries [59, 60]. Recent studies proved that early stage detection and phototreatment of bacteria in the bloodstream are available for MB-based multifunctional nanoplatforms [61]. By taking advantage of a specific antibody toward MDR *Salmonella* bacteria, MB molecules could be selectively delivered to *Salmonella* cells and lighten them with red fluorescence. Further introduction of core–shell Fe@Au nanoparticles into such multifunctional system ensures a successful magnetic separation of bacteria from whole blood sample, as well as a synergistic photodestruction of bacteria via Au-mediated photothermal therapy and MB-mediated PDT.

Notably, MB also reveals its potential in combating bacterial biofilm, a tricky problem for infection therapy. In clinic, bacterial infections often occur as biofilm forms in which pathogens are protected by extracellular polymeric substances (EPS) to prevent penetration and subsequent action of drugs [62]. In such cases, even very effective photosensitizers require high concentrations and light doses to eradicate biofilms. To address this issue, Sun et al. [63] have recently synthesized a kind of carboxymethyl chitosan nanoparticles in a facile and green way using MB as a cross-linking agent, as well as a fluorescent molecule and a photosensitizer for self-imaging PDT. In the weak acidic microenvironment of bacterial infection, the nanoparticles efficiently released MB molecules, allowing in situ fluorescence imaging of bacteria. When irradiation by a 650-nm laser at 202 mW cm^−2^ for 5 min, MB-contained nanoparticles exhibited potent photodynamic biofilm eradication. Such rapid sterilization exerted a significant effect on infection treatment, inflammation inhibition, and wound healing. Despite these advances, phenothiazinium photosensitizers used in current bacterial theranostics are still limited, mostly dependent on MB. Actually, it seems that MB is not the optimum option in the class of phenothiaziniums for aPDT. Various MB derivatives, such as new methylene blue, dimethyl methylene blue, and methylene green, have been developed with different functional substituents and performed increased aPDT efficacy than native MB molecule [64, 65]. Accelerating the exploitation of such novel-acting derivatives is strongly demanded for further phenothiazinium-based bacterial theranostics.

#### Others

Although a majority of researches are based on porphyrins, phthalocyanines, and phenothiaziniums, some special photosensitizers also show great promise in bacterial labeling and treatment. Fluorescent coumarin was recently transformed into bacteria-targeted theranostic application via covalently conjugating with cyanopyridinium units. By virtue of strong intramolecular charge transfer process and appropriate lipophilicity, the coumarin-mediated fluorophore showed intense near-infrared emission (675 nm) and was able to selectively anchor on Gram-positive bacteria. As a result, precise bacteria detection following by instant photodynamic sterilization was accomplished successfully [66]. It has been recently reported that indocyanine green (ICG), a FDA-approved near-infrared (NIR) fluorescent dye, has been progressed as photosensitizer for aPDT [67]. After encapsulation in cationic antimicrobial peptide-decorated manganese dioxide nanosystems, such integrated platform achieved a noninvasive and accurate diagnosis of bacterial osteomyelitis through ICG-mediated photoacoustic imaging and Mn^2+^-mediated *T*_1_ magnetic resonance imaging (MRI). Further in combination with low-dose gentamicin, a synergistic antibacterial effect of PDT and antibiotic was observed, effectively rescuing mice from bacterial bone infection.

As mentioned above, photosensitizers based on ACQ have indeed shown great promise for bacterial theranostics in vitro and in vivo. However, many of them are still far from ideal for clinical application. When designing photosensitizers, various factors should be considered. It is expected to endow them with a longer-wavelength absorption in the NIR region, which may offer better treatment on deeply seated diseases. Besides, an ideal photosensitizer should be safe enough, with low dark toxicity and short skin photosensitivity period. As for strong photochemical reactivity, a high extinction coefficient and a great quantum yield of ROS are indispensable. Finally, excellent specificity to infection sites and rapid clearance from normal tissues are also the important concern for clinical translation. If possible, the photosensitizer is preferable ease of administration via various routes. Suffice it to say, none of photosensitizers in current clinical use can be described as “optimal” or “ideal.” The pursuit of a novel alternative to simultaneously satisfy the crucial physical, chemical, and biological requirements is still ongoing.

### Photosensitizers Based on AIE

Surpassing traditional organic photosensitizers that usually suffer severe photobleaching and reduced ROS generation in the aggregated state, the newly developed photosensitizers with AIE characteristics are becoming attractive candidates for advanced bacterial theranostics. As a constructive effect, AIE makes it possible for aPDT to actively utilize the aggregation process, instead of passively working against it. Further careful molecular design endows the AIE photosensitizers with many fascinating features, including free of self-quenching, outstanding photostability, efficient ROS production, high signal-to-noise ratio, and low detection limit. Especially, the unique fluorescence “light-up” mode via flexible de-aggregation/aggregation processes qualifies AIE photosensitizers as a superior choice for optical imaging-guided aPDT [68, 69]. Despite these advantages, AIE photosensitizers are going through some challenges, such as low ROS production, poor bacterial targeting, and weak theranostic capability toward complicated bacterial infections. These severely restrict their further clinical application. How to circumvent such undesirable properties and improve the theranostics of AIE photosensitizers are in strong demand.

#### AIE Photosensitizers for High ROS Generation

**Fig. 5:**
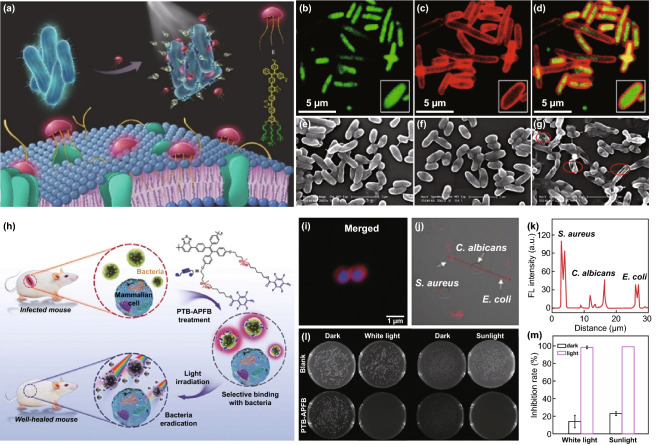
**a** Chemical structure and schematic illustration of membrane-anchoring AIE photosensitizer for bacteria membrane penetration. Confocal laser scanning microscopy (CLSM) images of *E. coli* treated with 10 μM membrane-anchoring AIE photosensitizer and then labeled in **b** false green color by DAPI, **c** red color by photosensitizer, **d** yellow color by overlay images. Scanning electron microscopy images of **e** blank *E. coli*, **f** *E. coli* + 10 μM AIE photosensitizer under dark condition, **g** *E. coli* + 10 μM AIE photosensitizer under white-light irradiation [71]. Copyright 2019 Wiley-VCH. **h** Chemical structure of PTB-APFB and its selective antibacterial application. **i** CLSM images of *S. aureus* incubated with PTB-APFB, followed with Hoechst 33342 co-staining. **j** CLSM images of PTB-APFB co-incubated with *S. aureus, E. coli*, and *C. albicans*. **k** Line scan analysis for the mixed sample. **l** Photographs of plate and **m** antibacterial activity of PTB-APFB against *S. aureus* in different conditions [72]. Copyright 2019 Wiley-VCH

It is noteworthy that the effectiveness of ROS generation by photosensitizers significantly depends on their capability of light absorption. Due to rotor structures of AIE photosensitizers, poor conjugation often plagues these molecules, yielding narrow absorption in the short wavelength region [70]. In this regard, how to qualify new AIE photosensitizers with broad absorption and large molar absorptivity is highly demanded for efficient bacterial theranostics. Fortunately, the introduction of electron-donor and electron-acceptor structural units into AIE backbone makes it available. The AIE photosensitizers with donor–π–acceptor (D–π–A) structures show great improvement in fluorescence imaging and ROS production as aggregates form. A representative example was reported by Liu’s group who designed the AIE photosensitizer with D (donor)-A’ (auxiliary acceptor)-π (π spacer)-A (acceptor) structure, while three cationic groups were involved in the molecular design for bacterial membrane anchoring (Fig. 5a–g) [71]. With a high ^1^O_2_ quantum yield of 0.48, this novel AIE photosensitizer showed efficient antibacterial performance toward both Gram-positive bacteria (*S. aureus*) and Gram-negative (*E. coli*). Further benefiting from the enhanced membrane interactions and broad absorption in the visible range, over 99.8% killing efficiency was observed for MRSA when they were exposed to 0.8 μM photosensitizer at a low white-light dose of 15 J cm^−2^. Similarly, Zhou et al. [72] reported an AIE-active polymer (PTB-APFB) for reliable bacterial imaging and eradication (Fig. 5h). Thanks to the AIE and donor–π–acceptor structure, this PTB-APFB polymer performed strong light-harvesting ability for fluorescence imaging toward different microorganisms, such as *S. aureus* (Gram-positive bacteria), *E. coli* (Gram-negative bacteria), and *Candida albicans* (fungi) (Fig. 5i–k). Compared to the common photosensitizer Ce6, the AIE polymer showed a higher production of ROS, resulting in significant inhibition on *S. aureus* growth upon light exposure (Fig. 5m).

#### AIE Photosensitizers for Bacterial Targeting

Targeted diagnosis and therapy highlight the need for pathogen-specific probes. Similar to ACQ photosensitizers, poor bacteria-specificity challenges photosensitizers based on AIE structure, making them unsuitable for accurately discerning bacterial infections, let alone determining the types of bacteria. Targeted drug delivery accelerates the emergence of new AIE theranostic options. By taking advantage of bacteria-piloting agents (e.g., cationic groups, peptides, monoclonal antibodies), these “light-up” photosensitizers can be tailed with selective labeling, distinguishing, and killing of bacteria [73–75]. For example, the AIE photosensitizer after conjugation with positively charged zinc(II)-dipicolylamine could specifically target to the negatively charged bacterial membrane via electrostatic interaction, but not to mammalian cells [76]. Similarly, a selective recognition, naked-eye visualization, and photodynamic inactivation of Gram-positive bacteria over Gram-negative bacteria were observed in vancomycin-functionalized AIE photosensitizer due to the excellent targeting of vancomycin to Gram-positive bacteria [77].

**Fig. 6:**
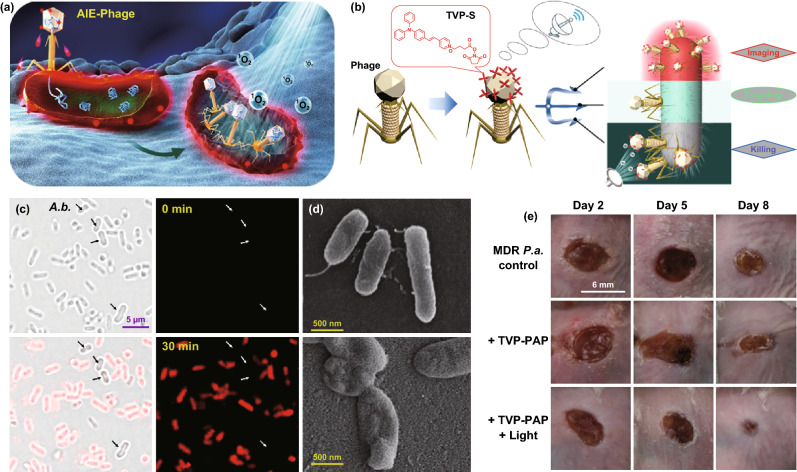
**a** Cartoon illumination of bacterial imaging by AIE fluorescence and the phage infections as well as the ROS generation of phage-modified AIEgens. **b** Synergistic effect of phage-modified AIEgens for bacteria-specific recognition, real-time fluorescent tracking, phage infection, and AIE-mediated aPDT. **c** Specificity evaluation of phage-modified AIEgens by fluorescence imaging of *Pseudomonas aeruginosa (P.a.)* and *Acinetobacter baumannii (A.b.)* indicated by arrows. **d** SEM imaging of bacteria before and after aPDT. **e** Photographs of wounds treated by phage-modified AIEgens and light irradiation for different time periods [78]. Copyright 2020 American Chemical Society

However, electrostatic interaction is not always effective for precise recognition and sterilization due to complicated biological environment. Continuous incidence of MDR “superbugs” poses a severe threat to antibiotics targeting. By contrast, bacteriophage shows specificity to their pathogenic hosts and can easily adapt to bacterial resistance with synchronous evolution. In this regard, the application of bacteriophage is expected to guide AIE for discriminative imaging and killing of a certain species of bacterium, with no apparent influence in the non-target bacteria or the normal mammalian cells (Fig. 6) [78]. After facile conjugation of bacteriophage with AIE photosensitizer, the generated bioconjugates perfectly preserved the properties of both phage and AIEgen, with pinpoint specificity for bacterial recognition and inherited fluorescence for real-time tracking of phage-bacterium interaction. Meanwhile, a synergistic antibacterial performance with superior efficiency was achieved by integrating phagotherapy and AIE-mediated PDT. Noteworthily, the selectivity of targetable bacteriophage-AIE probes is highly dependent on the specific recognition between susceptible hosts and phages. The natural host range of a bacteriophage isolate is limited, and the combined host ranges of all current investigated bacteriophage strains fail to encompass a sufficient fraction of the target bacteria of interest. Engineering bacteriophages with novel, designed-to-specification host ranges is indispensable to enlarge the role of phage-navigated AIE theranostics in the future.

In addition to bacterial recognition interaction, the metabolic biomolecular labeling technology has emerged as a powerful tool for pathogen targeting, because it is highly specific, unconstrained by MDR, and shows unique superiority in stability, convenience, and cost-effectiveness [79, 80]. This strategy has two steps: In the first step, a functionalized biomolecule is selectively metabolized by the target cells or living organisms to express specific reactive groups on their surfaces. Subsequently, probes decorated with corresponding reactive groups are introduced and then covalently conjugate to expressed reactive groups on the surface of target lesions. Recently, MIL-100 (Fe) nanoparticles have been examined as a carrier for precise delivery of 3-azido-d-alanine (d-AzAla), a metabolic labeling molecule for bacteria [81]. It was shown that the degradation of nanoparticles could be triggered within H_2_O_2_-oversecreted microenvironment of bacterial infections, thereby releasing d-AzAla to incorporate into the bacterial cell walls. Subsequently, ultrasmall photosensitizer nanoparticles with AIE characteristics were injected and attached to the modified bacteria via in vivo biorthogonal click chemistry. Benefiting from intense red fluorescence and strong photosensitizing capability of AIE photosensitizer, the MRSA-infected tissue was successfully imaged and relieved. This metabolic labeling method offers a new perspective for great improvement in bacteria-targeted AIE labeling, but the incorporation of exogenous reactive groups is challenged with large-scale applications and demands further technical optimization.

#### AIE Photosensitizers for Intracellular Bacteria

**Fig. 7:**
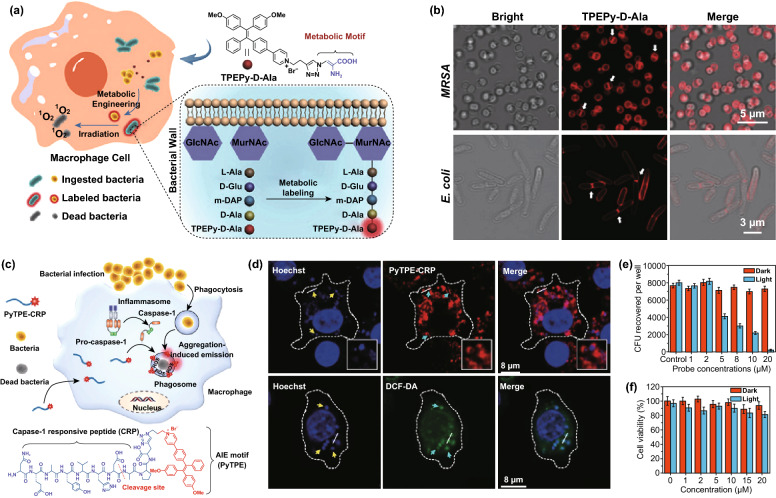
**a** Schematic illustration of TPEPy-D-Ala probe against intracellular bacterial. Involved in the metabolism of peptidoglycan, d-alanine allows the metabolic labeling of bacterial peptidoglycan by probe. **b** CLSM, bright field and merged images of TPEPy-D-Ala probe-treated MRSA and *E. coli* [84]. Copyright 2019 Wiley-VCH. **c** Schematic of PyTPE-CRP photosensitizer for intracellular bacterial diagnosis and elimination. **d** CLSM images of the PyTPE-CRP localization o in *S. aureus*-infected Raw 264.7 macrophages. White dashed lines indicate cellular outline. The *S. aureus* are shown with yellow arrows. Cyan arrows represent *S. aureus*-contained phagosomes. The DNA of bacterial cells and Raw 264.7 macrophages are labeled with blue Hoechst dye. **e** Intracellular survival of *S. aureus* in Raw 264.7 macrophages treated with PyTPE-CRP. **f** Viability of Raw 264.7 macrophages in the presence of PyTPE-CRP photosensitizer [85]. Copyright 2019 Wiley–VCH

Intracellular infections are another barrier hindering the effective management of bacterial theranostics. When infection does occur, bacteria are able to invade and survive inside mammalian cells, primarily the macrophage cells that are responsible for pathogen clearance [82]. Surviving in the host macrophages, these intracellular bacteria are protected from other immune attacks. In such case, the infected phagocytic cells not only fail to eliminate bacteria but may also act as “Trojan horses” for bacterial dissemination from the initial site, which could result in long-term chronic or recurrent infections [83]. With the aid of shielding effect from macrophage cells, the detection and ablation of hidden intracellular bacteria are typically difficult. Until now, there is limited success to address this issue, but AIE photosensitizers have taken an important step more recently. Based on the bacterial metabolic precursor of d-alanine and an AIE photosensitizer of pyridinium-substituted tetraphenylethylene, a dual-functional probe (TPEPy-d-Ala) was developed for visualization and in situ ablation of intracellular bacterial pathogens (Fig. 7a) [84]. Once metabolically incorporated into bacterial peptidoglycan, the AIE probe would trigger a fluorescence turn-on response, allowing us to clearly trace the intracellular bacteria in living macrophages (Fig. 7b). Subsequent light irradiation potently eradicated the labeled intracellular bacteria through singlet oxygen generation. Ultimately, the promising AIE photosensitizer achieved a low minimum inhibitory concentration of 20 ± 0.5 μg mL^−1^, much more potent than that of a commonly used antibiotic of vancomycin (100 μg mL^−1^). In a follow-up work, AIE photosensitizer (PyTPE-CRP) was tailored with caspase-1 responsive peptide, so that the bioconjugates can be efficiently cleaved, self-assemble, and activate bacterial theranostics on caspase-1 enzyme-enriched macrophage phagosomes (Fig. 7c) [85]. Owing to short lifetime of ROS and high bacterial targeting (Fig. 7d), such AIE probes are expected to accomplish highly potent bacteria killing while performing a negligible effect on macrophage cells (Fig. 7e, f).

So far, the application of AIE photosensitizers is still limited to animals and preclinical studies. To drive their clinical translation, many efforts are desirable to understand the ADME (absorption, distribution, metabolism, and excretion) of AIE photosensitizers, implementing a rational and systematic design methodology. Moreover, suffering from rotor structures and limited conjugation length, most of AIE photosensitizers exhibit short excitation wavelength from 390 to 550 nm, which severely restricts their tissue penetrability and is therefore not favorable for in vivo applications. The exploration of future AIE photosensitizers expectantly centers on elaborate molecular design to induce long wavelength absorption. Additionally, the introduction of upconversion nanoparticles (UCNPs) and the development of two-photon or multiphoton excited AIE photosensitizers will also widen their biomedical applications to deep-seated disease diagnosis and treatment.

### Photosensitizers Based on Nanoparticles

Photosensitizers based on nanomaterials are a type of nanosensitizers that have intrinsic photodynamic properties without needing guest sensitizers. Different from small molecular photosensitizers of ACQ or AIE, such nanosized photosensitizers are generally well dispersed and aggregate-free in water, which are conducive to elude the complement system and blood clearance [86]. Noticeably, the use of nanophotosensitizers provides an interesting opportunity where the nanomaterials become an active participant, instead of the passive vehicle for photosensitizer delivery. Thanks to their nanometric diameter and large surface area suitable for chemical functionalization, these nanoagents show an improvement of biocompatibility, biodistribution, and selectivity toward diseased tissues [87, 88]. In the past decade, the development of nanophotosensitizers has accelerated rapidly, with increasing number of reports on quantum dots, titanium dioxide, mesoporous silica, fullerene, gold, ruthenium, and upconversion nanoparticles, as well as two-dimension molybdenum disulfide and black phosphorus nanosheets [89]. Some of them hold great potential in bacterial theranostics due to innate imaging and PDT effects.

**Fig. 8:**
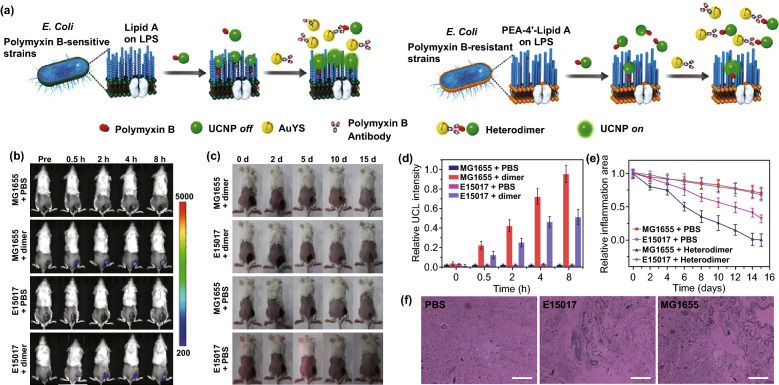
**a** Schematic illustration of Au YS and UCNP heterodimer for quantitative detection and imaging of drug-resistant bacteria. **b** Time-dependent in vivo UCL imaging before and after *i.v.* injection of Au YS-UCNP heterodimer into inoculation of polymyxin-B-sensitive strains (MG1655), polymyxin-B-resistant bacteria (E15017), and PBS control. **c** Representative photographs of mice for MG1655 or E15017 infection-bearing mice with Au YS-UCNP heterodimer or PBS treatment upon 532 nm irradiation. **d** Average UCL signals in the infected region at different time points in each group. **e** Relative inflammation area after heterodimer or PBS treatments for 15 days. **f** Hematoxylin and eosin (H&E) images of non-infected and infected sites. Scale bar: 100 μm [92]. Copyright 2018 Wiley-VCH

Quantum dots are semiconductor nanoparticles, which can transfer energy to surrounding oxygen in a similar manner to traditional photosensitizer. These small nanoparticles (size range of 1–6 nm) have constant composition, excellent photostability, high quantum yields, and tunable fluorescent emission properties [90]. Recently, a new type of two-photon excited quantum dots was constructed by doping graphene quantum dots (GQDs) with nitrogen and then functionalizing with an amino group (amino-N-GQDs) [91]. Compared with native GODs, these amino-N-GQDs exhibited desirable two-photon luminescence and stability, facilitating themselves as a favorable two-photon contrast agent for tracking and localizing bacteria in a three-dimension environment. Mediated by two-photon excitation, the MRSA bacteria were eliminated completely at an ultralow energy (239.36 nJ pixel^−1^, 12 s) in the NIR region (800 nm). Sun et al. [92] reported the use of gold nanoparticles as photosensitizer for the quantification and inactivation of polymyxin-B-resistant *E. coli* (Fig. 8a). In this approach, polymyxin-B-modified UCNPs and antipolymyxin-B-antibody coupled gold yolk–shell nanoparticles (Au YS NPs) were employed as the building blocks to implement bacteria-responsive heterodimer assembly. Depending on the different affinity of polymyxin B for sensitive and resistant strains, the UCNPs after co-incubation with bacteria showed poor accumulation in polymyxin-B-resistant species, revealing low intracellular upconversion luminescence (UCL) intensity. Correspondingly, the circular dichroism (CD) signal from Au YS NPs was dramatically increased in solution because the Au YS NPs could not enter the bacteria but formed heterodimer structure with the extracellular polymyxin-B-coupled UCNPs. Through the different CD (extracellular) and UCL (intracellular) intensities in both bacterial species, the polymyxin-B-resistant level of *E. coli* could be effectively detected with dual signals. Importantly, with the aid of assembled heterodimer platform, bacterially induced infection was successfully treated with UCL imaging-guided PDT in vivo (Fig. 8b–f).

Based on the above enlightenment, nanophotosensitizers with an ability of theranostic function are favored in bacterial theranostics. However, the reported examples are relatively few. Unlike the new emergence of bacterial theranostics, researches on the possible therapeutic application of nanophotosensitizers can date back to 1985 when the first work on photocatalytic disinfection using titanium dioxide nanoparticles (TiO_2_ NPs) was published. As a clinically approved photosensitizer, TiO_2_ NPs have long been manufactured and used world-wide against various microorganisms, such as *S. aureus*, *E. coli*, *Candida albicans*, and *Mycobacterium tuberculosis* [93]. Although pristine TiO_2_ NPs barely possess imaging capabilities, the fluorescent dye or magnetic resonance contrast agents, with the aid of nanotechnology, can be facilely labeled onto TiO_2_ NPs, facilitating them as theranostic agents for bacterial management. Compared to small molecular photosensitizers, these nanoparticles show better tunability, biological stability, and multifunctionality. However, many of the inorganic nanophotosensitizers have dose-dependent toxicity. When repeated medication, the cumulative risk is challengeable. Therefore, developing nanophotosensitizers with improved safety and biodegradability warrants substantial attention in future research.

## SDT-Mediated Bacterial Theranostics

Although aPDT has many inherent advantages and shows great promise to be a stand-alone modality in imaging and treating bacterial infections, its effectiveness against deep-seated diseases is limited due to the relatively shallow (< 1 cm) tissue penetration depth of light [94]. Compared to photoinduced antibacterial therapies that are mostly confined to skin lesion, aSDT takes advantage of superior tissue penetrability of ultrasonic wave, showing great feasibility in deeply seated infections. Currently, most studied sonosensitizers are based on tetrapyrrolic macrocycles, with emphasis on porphyrins. Several organic molecules (rose Bengal, cyanine, natural products), inorganic nanomaterials (titanium dioxide, black phosphorus, ZnO, Fe_3_O_4_, MnWO_*x*_), and their hybrids have also been proven as useful sonosensitizers for disease treatment [95]. Although sonotheranostics have proved remarkable and durable responses to antimicrobial stewardship, this approach is still at the preliminary stage and its related research is in infancy. Generally, complete eradication of bacterial infections is difficult by a single aSDT, partly because the low utilization efficiency of ultrasound energy causes limited cavitation effect, failing to sufficiently activate ROS generation for treatment. In addition, the high heterogeneity and adaptability of infection itself also pose great challenges for aSDT [96, 97]. Fortunately, several interesting approaches are being tried to maximize the efficacy of aSDT-assisted bacterial theranostics, which is originally considered as the basic classification profile in the following discussion.

### Bacteria-Specific Nanosystems

**Fig. 9:**
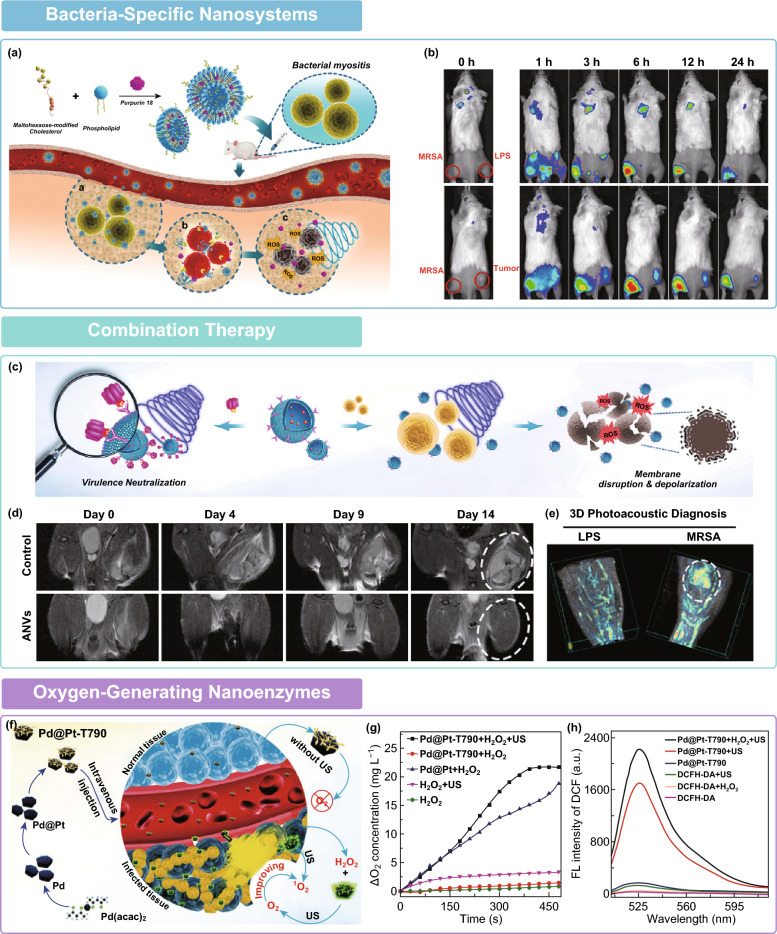
**a** Scheme illustration of MLP18 nanoliposomes for diagnosis and eradiation of bacterial infection. **b** NIR fluorescence images of MRSA and LPS infected mice or MRSA and 4T1 tumor-bearing mice model after MLP18 injection [99]. Copyright 2019 American Chemical Society. **c** Schematic illustration of the antivirulence and antibacterial mechanism of ANVs. **d** Representative magnetic resonance images of MRSA-infected mice within 14-day post-injection of ANVs. **e** 3D photoacoustic images of MRSA and LPS infected thighs from ANVs-treated mice [19]. Copyright 2019 Wiley-VCH. **f** Schematic illustration of Pd@Pt-T790 nanoenzyme for ultrasound-switchable oxygen generation and aSDT enhancement. The generation of **g** oxygen and **h** ROS by Pd@Pt-T790 nanoenzyme without or with ultrasound exposure [109]. Copyright 2020 American Chemical Society

Recently, the blooming advances in nanotechnology, particularly the development of targeted drug delivery, have substantially altered the traditional concepts of aSDT. A diversity of nano-assisted aSDT platforms have been elaborately engineered and deeply change the sonotheranostic behavior in different ways, including enhancing solubility of sonosensitizers, alleviating their auto-optical quenching, and modulating the in vivo fate [98]. Through exploiting bacteria-specific metabolism pathway, which is not present in the mammalian cells, there is a tremendous potential for implementing the bacteria-targeted sonotheranostics, even given it ability to precisely distinguish bacterial infections from sterile inflammatory diseases. As a typical paradigm, maltohexaose, a major substrate of bacteria-specific maltodextrin transport system, was decorated on the surface of nanoliposomes (Fig. 9a) [99]. The purpurin 18 payload in such maltohexaose-functionalized nanoliposomes (MLP18) acts as an excellent NIR fluorescence imaging/photoacoustic imaging (FLI/PAI) probe, as well as potent sonosensitizer. Upon ultrasound irradiation (1 MHz, 0.97 W cm^−2^, 5-min duration), the notorious clinical pathogens of MRSA and extended-spectrum β-lactamase *E. coli* (ESBL-EC) suffer from a series of biological changes, including wrinkled and collapsed cell walls, obvious leakage of the intracellular milieu, and impaired membrane integrity. Unlike clinically used tracers hampered by their short working window and low bacterial specificity, MLP18 can be used to monitor infection progress with longer optical period and for multiple times, while its highly selective FL/PA signal on the bacteria-infected site validated an accurate differentiation between infection foci and other pathological changes, such as sterile inflammation and cancer (Fig. 9b). With bacteria-triggered efficient release and internalization of sonosensitizer, an obvious sonodynamic elimination of MRSA myositis was accomplished in mice. Strikingly, this sonolethal action was broad-spectrum regardless of bacterial species and drug-resistance. Further considering the excellent clinical translatability of nanoliposomes which are responsible for more than 50% of the total nanoformulations commercially available, the MLP18 with great biocompatibility and biodegradability serve as a promising sonotheranostic platform against MDR bacteria in the areas of healthcare.

### Combination Therapy

Another compelling strategy is combining aSDT with other therapeutic modalities for synergistic antibacterial management. Such combinations mainly focus on enhancing the bacterial sensitivity to SDT, as well as breaking the cytoprotection induced by SDT-survived bacterial cells. Considering the controllability of ultrasound irradiation, only lesion site receives ultrasound exposure. The overlapping toxicities from combination therapy are therefore expectantly limited to a finite area, which avoids the potential threats to systemic safety [100]. Such effect is of great importance in infection patients, especially those who are too debilitated or elder to tolerate more intensive therapeutic regimens. More importantly, because of the unique non-specific therapeutic mechanism, aSDT allows safe combination therapy without little risk of crossed multidrug resistance. For example, aSDT has been widely reported to integrate with aPDT based on the fact that most of sonosensitizers are concurrently the photosensitizers [101]. Given this, a photo/sonosensitizer hematoporphyrin monomethyl ether was modified on the surface of Fe_3_O_4_-coated UCNPs (Fe@UCNP-HMME) [102]. Compared with PDT alone or SDT alone, the sonophotodynamic therapy (1 W cm^−2^ of laser, 2 W cm^−2^ of ultrasound, 10 min) achieved higher ROS production and antibacterial effectiveness, revealing a 100% bacterial killing on MRSA and ESBL-EC in vitro. Besides the excellent therapeutic capability, an in vivo dual-modality imaging, including MRI and UCL imaging, was also successfully performed. Such unique combination solves the ROS limitation of aSDT and significantly enhances the therapy efficiency through dual application of ROS-producing PDT and SDT.

Antivirulence-involved combination therapy provides another alluring way to extend the usefulness of current aSDT in an era of MDR bacterial sonotheranostics. During infection initiation and progression, bacteria often utilize multiple and redundant pathogenic mechanisms; thereinto, secreted toxins play a crucial role in the pathogenesis of many medically important bacteria. Different from direct bacteriostatic and bactericidal mechanisms like aSDT, antivirulence therapy selectively disarms bacterial pathogenicity via neutralizing their virulence determinants. This “disarm-not kill” approach aims to combat immunosuppression and protect innate immune defense from virulence subversion [103]. When combination with aSDT, such complementary regimen that allows simultaneous attack on both bacteria and bacteria-associated virulence is superior to remarkably improve the probability of treatment success, even such difficult-to-manipulate organisms as MRSA were proved tractable [19]. As a proof-of-principle, a neutralizing monoclonal antibody (mAb) was genetically displayed on the exterior of cell membrane nanovesicles to capture alpha-toxin, the key virulence factor of MRSA bacteria. After sonosensitizers encapsulation, such nanoplatform (ANVs) facilely bridges aSDT with antivirulence-mediated immunotherapy. The sonosensitizers upon ultrasound activation (1 MHz, 0.97 W cm^−2^, 50% duty cycle, 8 min) efficiently generate ROS to kill bacteria, meanwhile, pathogenic toxin is robustly cleared by neutralizing mAb (Fig. 9c). As a result, the mice with MRSA myositis were successfully rescued by this sonoimmunotherapy, revealing a complete eradication of the muscular abscess in MRI monitoring (Fig. 9d). Benefiting from the exquisitely specific antibody–ligand interaction and inherent luminescent features of sonosensitizer, the mAb-piloting sonoimmune combination also offers precise imaging diagnosis of bacterial infection (Fig. 9e). Notably, such antibiotic-free combination treatment may impose little to no selective pressure for resistance, leaves the host commensal microbiota unscathed, and is rarely cross-resistant with each other. In addition to sonophotodynamic therapy and sonoimmunotherapy, the aSDT combined with photothermal therapy also showed superior antibacterial performance, guaranteeing a potent treatment of bacterial infected bone implants [104]. Considering these exciting results, the aSDT-mediated combination therapy may represent a highly promising approach for effective imaging and complete eradication of MDR bacterial infections.

### Oxygen-Generating Nanoenzymes

Oxygen deficiency is another inherent limitation governing the outcome of aSDT. Unequivocally, aSDT is contingent upon the sustained ROS generation. This process is closely related to the oxygen content of infection region. However, most bacterial infections develop a microenvironment of severe hypoxia, and this is more deteriorated with the infection progression [105]. Such hypoxic tissues put them beyond the reach of most therapeutic protocols. Troublesomely, aSDT, on the other hand, is restricted more seriously, because the rapid consumption of oxygen in sonodynamic action greatly aggravates acute hypoxia, further inhibiting the therapeutic process. Recent advances in nanotechnology and material chemistry have provided new design rationales for aSDT in modulating micro-environmental hypoxia and favoring sonotheranostics. These exquisite sonodynamic platforms are mostly established by integrating inorganic nanoenzymes (e.g., catalase [106], MnO_2_ [107]) and can efficiently convert endogenous H_2_O_2_ to O_2_ in an enzyme-catalyzed manner. With the aid of oxygen generation, the hypoxia level of infection microenvironment is effectively alleviated, which potentially favors the subsequent ROS production for therapeutic enhancement [108]. More interestingly, researches are now seeking to conceive more sophisticated and ingenious nanoenzymes to regulate hypoxia by means of, for example, endowing them with stimuli-responsive catalytic activity so as to precisely control enzymatic performance and decrease side effects. A representative paradigm is sonosensitizer T790 modified Pd@Pt nanosystems [109], in which sonosensitizer attachment significantly blocks the catalase-like activity of Pd@Pt nanosheets, but the enzyme activity can be effectively restored upon ultrasound irradiation (1 MHz, 0.97 W cm^−2^, 50% duty cycle, 8 min) to catalyze oxygen generation (Fig. 9f–h). Under the guidance of triple-modal imaging techniques, including FLI, PAI, and computed tomography (CT) imaging, infection site was well located by sonotheranostic nanoenzyme so that ultrasound irradiation can be accurately applied to realize complete eradication of bacterial myositis in mice. Such “blocking and activating” catalysis enables the precise regulation of oxygen and augments ROS production. This is particularly important for reducing the potential toxicity of nanoenzymes on normal tissues and promises to realize active, controllable, and disease loci-specific catalytic behavior.

## Conclusion and Perspectives

Injudicious use and inordinate prescription of antibiotics has driven the global spreading of MDR pathogens, greatly challenging the diagnostic and therapeutic techniques in modern medicine. Recent advances in aPDT/SDT provide new opportunities for the management of MDR bacterial infections, especially in the theranostic domain. Till now, a number of photosensitizers have been developed for bacterial theranostics, which are not only traditional ACQ photosensitizers, but also novel-acting AIEgens and nanoparticles. Starting from aPDT, the emerging aSDT circumvents the penetration depth limitation of aPDT, showing great potential in the imaging and treatment of deep-seated bacterial infections. These theranostic approaches allow personalized antibacterial stewardship to promptly implement appropriate treatment regimen. Although more targeted and effective, their therapeutic efficacies are still not satisfactory enough to fully accomplish a timely and complete infection eradication. Fortunately, several elegant solutions have been proposed, including employing nanotechnology to improve in vivo fate of sensitizers, introducing metabolic biomolecular labeling or bacteria-specific ligands for precise sensitizer delivery, integrating aPDT/SDT with other treatments, and exploiting oxygen-generating nanoenzymes to relieve hypoxia barrier.

Moving forward, the generation efficacy of ROS is a key to aPDT/SDT success. Through elaborate optimization of known sensitizers and development of novel-efficient sensitizers, these photo/sonotheranostic approaches are promising to highly produce ROS, especially the most destructive hydroxyl radical. Moreover, to fulfill the potential role of aPDT/SDT as weapons countering difficult-to-treat infections, active immunotherapy can serve as a complementing strategy. In general, infections becoming fatal always involve two major factors: (1) pathogenic activity by bacteria and (2) prolonged inflammation caused by the body’s immune system. Host-directed immunotherapy is expected to regulate host defense mechanisms, balance immune reactivity at sites of pathology, and ultimately allow the immune system to clear bacteria and repair tissue [110]. Additionally, the next-generation photo/sonotheranostic platforms also underscore the importance of siderophore-mediated “Trojan horse” approach for sensitizers delivery, radiolabeled aPDT/SDT systems to integrate clinically available nuclear imaging and radiotherapy, as well as more in-depth multidisciplinary collaboration among chemists, biologists, material scientists, and clinicians.

## References

[1] Jung SH, Ryu CM, Kim JS (2019). Bacterial persistence: fundamentals and clinical importance. J. Microbiol..

[2] Trotter AJ, Aydin A, Strinden MJ, O’Grady J (2019). Recent and emerging technologies for the rapid diagnosis of infection and antimicrobial resistance. Curr. Opin. Microbiol..

[3] J. Neill, Tackling drug-resistant infections globally: final report and recommendations. The review on antimicrobial resistance. (2016). http://amr-review.org/sites/default/files/160525_Final%20paper_with%20cover.pdf

[4] Loonen AJ, Wolffs PF, Bruggeman CA, van den Brule AJ (2014). Developments for improved diagnosis of bacterial bloodstream infections. Eur. J. Clin. Microbiol. Infect. Dis..

[5] Naqvi SAR, Drlica K (2017). Fluoroquinolones as imaging agents for bacterial infection. Dalton Trans..

[6] Dutta J, Naicker T, Ebenhan T, Kruger HG, Arvidsson PI (2017). Synthetic approaches to radiochemical probes for imaging of bacterial infections. Eur. J. Med. Chem..

[7] Livermore DM, John W (2013). Revolutionising bacteriology to improve treatment outcomes and antibiotic stewardship. Infect. Chemother..

[8] Sun J, Wang B, Warden AR, Cui D, Ding X (2019). Overcoming multidrug-resistance in bacteria with a two-step process to repurpose and recombine established drugs. Anal. Chem..

[9] Xie L, Pang X, Yan X, Dai Q, Lin H (2020). Photoacoustic imaging-trackable magnetic microswimmers for pathogenic bacterial infection treatment. ACS Nano.

[10] Zhi X, Liu Y, Lin L, Yang M, Zhang L (2019). Oral pH sensitive GNS@ab nanoprobes for targeted therapy of *Helicobacter pylori* without disturbance gut microbiome. Nanomedicine.

[11] Pan CL, Chen MH, Tung FI, Liu TY (2017). A nanovehicle developed for treating deep-seated bacteria using low-dose X-ray. Acta Biomater..

[12] Relman DA (2017). Microbiota: a high-pressure situation for bacteria. Nature.

[13] Mahmoudi H, Bahador A, Pourhajibagher M, Alikhani MY (2018). Antimicrobial photodynamic therapy: an effective alternative approach to control bacterial infections. J. Lasers Med. Sci..

[14] Cieplik F, Deng D, Crielaard W, Buchalla W, Hellwig E (2018). Antimicrobial photodynamic therapy - what we know and what we don’t. Crit. Rev. Microbiol..

[15] Serpe L, Giuntini F (2015). Sonodynamic antimicrobial chemotherapy: first steps towards a sound approach for microbe inactivation. J. Photochem. Photobiol., B.

[16] Dharmaraja AT (2017). Role of reactive oxygen species (ROS) in therapeutics and drug resistance in cancer and bacteria. J. Med. Chem..

[17] Franco TPM, Dos Santos APP, Canabarro A (2019). The effects of repeated applications of antimicrobial photodynamic therapy in the treatment of residual periodontal pockets: a systematic review. Lasers Med. Sci..

[18] Mao C, Xiang Y, Liu X, Cui Z, Yang X (2018). Repeatable photodynamic therapy with triggered signaling pathways of fibroblast cell proliferation and differentiation to promote bacteria-accompanied wound healing. ACS Nano.

[19] Pang X, Liu X, Cheng Y, Zhang C, Ren E (2019). Sono-immunotherapeutic nanocapturer to combat multidrug-resistant bacterial infections. Adv. Mater..

[20] Alves E, Faustino MA, Neves MG, Cunha A, Tome J (2014). An insight on bacterial cellular targets of photodynamic inactivation. Future Med. Chem..

[21] Foote CS (1991). Definition of type I and type II photosensitized oxidation. Photochem. Photobiol..

[22] Wu J, Nyborg WL (2008). Ultrasound, cavitation bubbles and their interaction with cells. Adv. Drug Deliv. Rev..

[23] Leighton TG, Pickworth MJW, Walton AJ, Dendy PP (1988). Studies of the cavitational effects of clinical ultrasound by sonoluminescence: 1. Correlation of sonoluminescence with the standing wave pattern in an acoustic field produced by a therapeutic unit. Phys. Med. Biol..

[24] Pan X, Wang H, Wang S, Sun X, Wang L (2018). Sonodynamic therapy(SDT): a novel strategy for cancer nanotheranostics. Sci. China. Life Sci..

[25] Leighton TG (2007). What is ultrasound?. Prog. Biophys. Mol. Biol..

[26] Bemporad D, Luttmann C, Essex JW (2004). Computer simulation of small molecule permeation across a lipid bilayer: dependence on bilayer properties and solute volume, size, and cross-sectional area. Biophys. J..

[27] Liu Y, Qin R, Zaat S, Breukink E, Heger M (2015). Antibacterial photodynamic therapy: overview of a promising approach to fight antibiotic-resistant bacterial infections. J. Clin. Transl. Res..

[28] Chilakamarthi U, Giribabu L (2017). Photodynamic therapy: past, present and future. Chem. Rec..

[29] Silhavy TJ, Kahne D, Walker S (2010). The bacterial cell envelope. Cold Spring Harb. Perspect. Biol..

[30] Pattison DI, Rahmanto AS, Davies MJ (2012). Photo-oxidation of proteins. Photochem. Photobiol. Sci..

[31] Jori G, Fabris C, Soncin M, Ferro S, Coppellotti O (2006). Photodynamic therapy in the treatment of microbial infections: Basic principles and perspective applications. Lasers Surg. Med..

[32] Chen C, Wang J, Li X, Liu X, Han X (2017). Recent advances in developing photosensitizers for photodynamic cancer therapy. Comb. Chem. High Throughput Screen.

[33] Tang BZ, Zhao Z, Zhang H, Lam JWY (2020). Aggregation-induced emission: new vistas at aggregate level. Angew. Chem. Int. Ed..

[34] Misba L, Zaidi S, Khan AU (2017). A comparison of antibacterial and antibiofilm efficacy of phenothiazinium dyes between Gram positive and Gram negative bacterial biofilm. Photodiagnosis Photodyn. Ther..

[35] García I, Ballesta S, Gilaberte Y, Rezusta A, Pascual Á (2015). Antimicrobial photodynamic activity of hypericin against methicillin-susceptible and resistant *Staphylococcus aureus* biofilms. Future Microbiol..

[36] Halili F, Arboleda A, Durkee H, Taneja M, Miller D (2016). Rose bengal- and riboflavin-mediated photodynamic therapy to inhibit methicillin-resistant *Staphylococcus aureus* keratitis isolates. Am. J. Ophthalmol..

[37] Maisch T (2007). Anti-microbial photodynamic therapy: useful in the future?. Lasers Med. Sci..

[38] Ghorbani J, Rahban D, Aghamiri S, Teymouri A, Bahador A (2018). Photosensitizers in antibacterial photodynamic therapy: an overview. Laser Ther..

[39] Gomes A, Neves M, Cavaleiro JAS (2018). Cancer, photodynamic therapy and porphyrin-type derivatives. An. Acad. Bras. Cienc..

[40] Alves E, Costa L, Carvalho CM, Tomé JOP, Faustino MA (2009). Charge effect on the photoinactivation of Gram-negative and Gram-positive bacteria by cationicmeso-substituted porphyrins. BMC Microbiol..

[41] Prasanth CS, Karunakaran SC, Paul AK, Kussovski V, Mantareva V (2014). Antimicrobial photodynamic efficiency of novel cationic porphyrins towards periodontal Gram-positive and Gram-negative pathogenic bacteria. Photochem. Photobiol..

[42] Zhao Z, Yan R, Wang J, Wu H, Wang Y (2017). A bacteria-activated photodynamic nanosystem based on polyelectrolyte-coated silica nanoparticles. J. Mater. Chem. B.

[43] Mazrad ZAI, Choi CA, Kwon YM, In I, Lee KD (2017). Design of surface-coatable NIR-responsive fluorescent nanoparticles with pei passivation for bacterial detection and killing. ACS Appl. Mater. Interfaces.

[44] Mai B, Jia M, Liu S, Sheng Z, Li M (2020). Smart hydrogel-based DVDMS/bFGF nanohybrids for antibacterial phototherapy with multiple damaging sites and accelerated wound healing. ACS Appl. Mater. Interfaces.

[45] Jiang Z, Vasil AI, Hale J, Hancock REW, Hodges RS (2009). Effects of net charge and the number of positively charged residues on the biological activity of amphipathic α-helical cationic antimicrobial peptides. Biopolymers.

[46] Jia HR, Zhu YX, Chen Z, Wu FG (2017). Cholesterol-assisted bacterial cell surface engineering for photodynamic inactivation of Gram-positive and Gram-negative bacteria. ACS Appl. Mater. Interfaces.

[47] Liu F, Soh Yan Ni A, Lim Y, Mohanram H, Bhattacharjya S (2012). Lipopolysaccharide neutralizing peptide-porphyrin conjugates for effective photoinactivation and intracellular imaging of Gram-negative bacteria strains. Bioconjug. Chem..

[48] Zhou J, Qi G-B, Wang H (2016). A purpurin-peptide derivative for selective killing of Gram-positive bacteria via insertion into cell membrane. J. Mater. Chem. B..

[49] Tang J, Chu B, Wang J, Song B, Su Y (2019). Multifunctional nanoagents for ultrasensitive imaging and photoactive killing of Gram-negative and Gram-positive bacteria. Nat. Commun..

[50] Tim Maisch (2015). Strategies to optimize photosensitizers for photodynamic inactivation of bacteria. J. Photochem. Photobiol., B.

[51] Brasseur N, Ouellet R, La Madeleine C, van Lier JE (1999). Water-soluble aluminium phthalocyanine-polymer conjugates for PDT: photodynamic activities and pharmacokinetics in tumour-bearing mice. Br. J. Cancer.

[52] Barthel M, Dini D, Vagin S, Hanack M (2002). An easy route for the synthesis of new axially substituted titanium(IV) phthalocyanines. European J. Org. Chem..

[53] Colussi VC, Feyes DK, Mulvihill JW, Li YS, Kenney ME (1999). Phthalocyanine 4 (Pc 4) photodynamic therapy of human OVCAR-3 tumor xenografts. Photochem. Photobiol..

[54] Grüner MC, Niemann S, Faust A, Strassert CA (2018). Axially decorated Si^IV^-phthalocyanines bearing mannose- or ammonium-conjugated siloxanes: comparative bacterial labeling and photodynamic inactivation. Photochem. Photobiol..

[55] Claessens CG, González-Rodríguez D, Torres T (2002). Subphthalocyanines: singular nonplanar aromatic compounds-synthesis, reactivity, and physical properties. Chem. Rev..

[56] Hota R, Baek K, Yun G, Kim Y, Jung H (2013). Self-assembled, covalently linked, hollow phthalocyanine nanospheres. Chem. Sci..

[57] Roy I, Shetty D, Hota R, Baek K, Kim J (2015). A multifunctional subphthalocyanine nanosphere for targeting, labeling, and killing of antibiotic-resistant bacteria. Angew. Chem. Int. Ed..

[58] Wainwright M, Crossley KB (2012). Methylene blue - a therapeutic dye for all seasons?. J. Chemother..

[59] Prażmo EJ, Mielczarek A, Kwaśny M, Łapiński M (2016). Photodynamic therapy as a promising method used in the treatment of oral diseases. Adv. Clin. Exp. Med..

[60] Fontana CR, Abernethy AD, Som S, Ruggiero K, Soukos NS (2009). The antibacterial effect of photodynamic therapy in dental plaque-derived biofilms. J. Periodontal Res..

[61] Dai X, Fan Z, Lu Y, Ray PC (2013). Multifunctional nanoplatforms for targeted multidrug-resistant-bacteria theranostic applications. ACS Appl. Mater. Interfaces.

[62] Flemming HC, Wingender J, Szewzyk U, Steinberg P, Rice SA (2016). Biofilms: an emergent form of bacterial life. Nat. Rev. Microbiol..

[63] Sun L, Jiang W, Zhang H, Guo Y, Chen W (2019). Photosensitizer-loaded multifunctional chitosan nanoparticles for simultaneous in situ imaging, highly efficient bacterial biofilm eradication, and tumor ablation. ACS Appl. Mater. Interfaces.

[64] Gollmer A, Felgenträger A, Bäumler W, Maisch T, Späth A (2015). A novel set of symmetric methylene blue derivatives exhibits effective bacteria photokilling – a structure–response study. Photochem. Photobiol. Sci..

[65] Felgenträger A, Maisch T, Dobler D, Späth A (2013). Hydrogen bond acceptors and additional cationic charges in methylene blue derivatives: photophysics and antimicrobial efficiency. Biomed. Res. Int..

[66] Zhao N, Li Y, Yin W, Zhuang J, Jia Q (2020). Controllable coumarin-based NIR fluorophores: selective subcellular imaging, cell membrane potential indication, and enhanced photodynamic therapy. ACS Appl. Mater. Interfaces.

[67] Lu X, Chen R, Lv J, Xu W, Chen H (2019). High-resolution bimodal imaging and potent antibiotic/photodynamic synergistic therapy for osteomyelitis with a bacterial inflammation-specific versatile agent. Acta Biomater..

[68] Yuan Y, Liu B (2017). Visualization of drug delivery processes using aiegens. Chem. Sci..

[69] Zhuang J, Yang H, Li Y, Wang B, Li N (2020). Efficient photosensitizers with aggregation-induced emission characteristics for lysosome- and Gram-positive bacteria-targeted photodynamic therapy. Chem. Commun..

[70] Hu F, Huang Y, Zhang G, Zhao R, Yang H (2014). Targeted bioimaging and photodynamic therapy of cancer cells with an activatable red fluorescent bioprobe. Anal. Chem..

[71] Chen H, Li S, Wu M, Huang Z (2020). Membrane-anchoring photosensitizer with aggregation-induced emission characteristics for combating multidrug-resistant bacteria. Angew. Chem. Int. Ed..

[72] Zhou T, Hu R, Wang L, Qiu Y, Zhang G (2020). An AIE-active conjugated polymer with high ROS-generation ability and biocompatibility for efficient photodynamic therapy of bacterial infections. Angew. Chem. Int. Ed..

[73] Gao S, Yan X, Xie G, Zhu M, Ju X (2019). Membrane intercalation-enhanced photodynamic inactivation of bacteria by a metallacycle and TAT-decorated virus coat protein. Proc. Natl. Acad. Sci. U. S. A..

[74] Li Q, Li Y, Min T, Gong J, Du L (2019). Time-dependent photodynamic therapy for multiple targets: a highly efficient AIE-active photosensitizer for selective bacterial elimination and cancer cell ablation. Angew. Chem. Int. Ed..

[75] Kang M, Zhou C, Wu S, Yu B, Zhang Z (2019). Evaluation of structure-function relationships of aggregation-induced emission luminogens for simultaneous dual applications of specific discrimination and efficient photodynamic killing of Gram-positive bacteria. J. Am. Chem. Soc..

[76] Gao M, Hu Q, Feng G, Tomczak N, Liu R (2015). A multifunctional probe with aggregation-induced emission characteristics for selective fluorescence imaging and photodynamic killing of bacteria over mammalian cells. Adv. Healthc. Mater..

[77] Feng G, Yuan Y, Fang H, Zhang R, Xing B (2015). A light-up probe with aggregation-induced emission characteristics (AIE) for selective imaging, naked-eye detection and photodynamic killing of Gram-positive bacteria. Chem. Commun..

[78] He X, Yang Y, Guo Y, Lu S, Du Y (2020). Phage-guided targeting, discriminative imaging, and synergistic killing of bacteria by AIE bioconjugates. J. Am. Chem. Soc..

[79] Mahlapuu M, Håkansson J, Ringstad L, Björn C (2016). Antimicrobial peptides: an emerging category of therapeutic agents. Front. Cell. Infect Microbiol..

[80] Saylor C, Dadachova E, Casadevall A (2009). Monoclonal antibody-based therapies for microbial diseases. Vaccine.

[81] Mao D, Hu F, Ji S, Wu W (2018). Metal–organic-framework-assisted in vivo bacterial metabolic labeling and precise antibacterial therapy. Adv. Mater..

[82] Gresham HD, Lowrance JH, Caver TE, Wilson BS, Cheung AL (2000). Survival of *Staphylococcus aureus* inside neutrophils contributes to infection. J. Immunol..

[83] Thwaites GE, Gant V (2011). Are bloodstream leukocytes Trojan horses for the metastasis of *Staphylococcus aureus*?. Nat. Rev. Microbiol..

[84] Hu F, Qi G, Mao D, Zhou S (2019). Visualization and *in situ* ablation of intracellular bacterial pathogens through metabolic labeling. Angew. Chem. Int. Ed..

[85] Qi G, Hu F, Shi L, Wu M (2019). An AIEgen-peptide conjugate as a phototheranostic agent for phagosome-entrapped bacteria. Angew. Chem. Int. Ed..

[86] Abrahamse H, Kruger CA, Kadanyo S, Mishra A (2017). Nanoparticles for advanced photodynamic therapy of cancer. Photomed. Laser Surg..

[87] Lucky SS, Soo KC, Zhang Y (2015). Nanoparticles in photodynamic therapy. Chem. Rev..

[88] Li C, Wang X, Chen F, Zhang C, Zhi X (2013). The antifungal activity of graphene oxide-silver nanocomposites. Biomaterials.

[89] Krajczewski J, Rucińska K, Townley HE, Kudelski A (2019). Role of various nanoparticles in photodynamic therapy and detection methods of singlet oxygen. Photodiagnosis Photodyn. Ther..

[90] Pleskova S, Mikheeva E, Gornostaeva E (2018). Using of quantum dots in biology and medicine. Adv. Exp. Med. Biol..

[91] Kuo WS, Shao YT, Huang KS, Chou TM, Yang CH (2018). Antimicrobial amino-functionalized nitrogen-doped graphene quantum dots for eliminating multidrug-resistant species in dual-modality photodynamic therapy and bioimaging under two-photon excitation. ACS Appl. Mater. Interfaces.

[92] Sun M, Qu A, Hao C, Wu X, Xu L (2018). Chiral upconversion heterodimers for quantitative analysis and bioimaging of antibiotic-resistant bacteria *in vivo*. Adv. Mater..

[93] Foster HA, Ditta IB, Varghese S, Steele A (2011). Photocatalytic disinfection using titanium dioxide: spectrum and mechanism of antimicrobial activity. Appl. Microbiol. Biotechnol..

[94] Mallidi S, Anbil S, Bulin AL, Obaid G, Ichikawa M (2016). Beyond the barriers of light penetration: strategies, perspectives and possibilities for photodynamic therapy. Theranostics.

[95] Son S, Kim JH, Wang X, Zhang C, Yoon SA (2020). Multifunctional sonosensitizers in sonodynamic cancer therapy. Chem. Soc. Rev..

[96] Lammas DA, De Heer E, Edgar JD, Novelli V, Ben-Smith A (2010). Heterogeneity in the granulomatous response to mycobacterial infection in patients with defined genetic mutations in the interleukin 12-dependent interferon-gamma production pathway. Int. J. Exp. Pathol..

[97] de Almeida DF, Hungria M, Guimarães CT, Antônio RV, Almeida FC (2003). The complete genome sequence of *Chromobacterium violaceum* reveals remarkable and exploitable bacterial adaptability. Proc. Natl. Acad. Sci. U. S. A..

[98] Herrmann IK (2015). How nanotechnology-enabled concepts could contribute to the prevention, diagnosis and therapy of bacterial infections. Crit. Care.

[99] Pang X, Xiao Q, Cheng Y, Ren E, Lian L (2019). Bacteria-responsive nanoliposomes as smart sonotheranostics for multidrug resistant bacterial infections. ACS Nano.

[100] Yang B, Chen Y, Shi J (2019). Reactive oxygen species (ROS)-based nanomedicine. Chem. Rev..

[101] Sadanala KC, Chaturvedi PK, Seo YM, Kim JM, Jo YS (2014). Sono-photodynamic combination therapy: a review on sensitizers. Anticancer Res..

[102] Zongfang W, Chengcheng L, Yiming Z, Min H, Dandan M (2019). Photomagnetic nanoparticles in dual-modality imaging and photo-sonodynamic activity against bacteria. Chem. Eng. J..

[103] Fang RH, Luk BT, Hu CMJ, Zhang L (2015). Engineered nanoparticles mimicking cell membranes for toxin neutralization. Adv. Drug Deliv. Rev..

[104] Su K, Tan L, Liu X, Cui Z, Zheng Y (2020). Rapid photo-sonotherapy for clinical treatment of bacterial infected bone implants by creating oxygen deficiency using sulfur doping. ACS Nano.

[105] Benoit DS, Koo H (2016). Targeted, triggered drug delivery to tumor and biofilm microenvironments. Nanomedicine.

[106] Li G, Wang S, Deng D, Xiao Z, Dong Z (2020). Fluorinated chitosan to enhance transmucosal delivery of sonosensitizer-conjugated catalase for sonodynamic bladder cancer treatment post-intravesical instillation. ACS Nano.

[107] Ding B, Zheng P, Ma P, Lin J (2020). Manganese oxide nanomaterials: synthesis, properties, and theranostic applications. Adv. Mater..

[108] Zhu P, Chen Y, Shi J (2018). Nanoenzyme-augmented cancer sonodynamic therapy by catalytic tumor oxygenation. ACS Nano.

[109] Sun D, Pang X, Cheng Y, Ming J, Xiang S (2020). Ultrasound-switchable nanozyme augments sonodynamic therapy against multidrug-resistant bacterial infection. ACS Nano.

[110] Kaufmann SHE (2017). Dorhoi1 A, Hotchkiss RS, Bartenschlager R (2017) Host-directed therapies for bacterial and viral infections. Nat. Rev..

